# Increasing trend of exclusive breastfeeding over 12 years period (2002–2014) among women in Moshi, Tanzania

**DOI:** 10.1186/s12884-018-2104-7

**Published:** 2018-12-04

**Authors:** Ola Jahanpour, Sia E. Msuya, Jim Todd, Babill Stray-Pedersen, Melina Mgongo

**Affiliations:** 1 Institute of Public Health, Department of Epidemiology and Biostatistics, Kilimanjaro Christian Medical University College (KCMUCo), P.O. Box 2240, Moshi, Tanzania; 2 Department of Community Medicine, Kilimanjaro Christian Medical Centre (KCMC), Moshi, Tanzania; 3 Better Health for African Mother and Child (BHAMC), Moshi, Tanzania; 4 London School of Hygiene and Tropical Medicine, bloomsbury, UK; 5 Division of Women, Oslo University Hospital, University of Oslo, Oslo, Norway; 6 Institute of Clinical Medicine, University of Oslo, Oslo, Norway

**Keywords:** Trend, Exclusive breastfeeding, Regional level, Wealth quintile, HIV status, Tanzania

## Abstract

**Background:**

The World Health Organization has recommended that all infants under 6 months should be exclusively breastfed. An understanding of the trend of exclusive breastfeeding (EBF) over years and over smaller geographical areas is crucial to monitor the progress made in improving the proportions of infants’ EBF.

**Methods:**

Data on infant feeding practices on 2315 mother-infant pairs from 2002 to 2014 were extracted from cohorts of women who delivered in the Moshi Municipality. Descriptive statistics were used to establish the trend of EBF up to 1, 3 and 6 months across waves (2002/2004 = wave I, 2005/2012 = wave II and 2013/2014 = wave III), to relate EBF up to 6 months to wealth quintiles and to HIV status of mothers.

**Results:**

The number of mothers in waves I, II and III were 1656 (71.5%), 256 (11.1%) and 403(17.4%) respectively. The percentages of EBF up to 6 months increased from 5.5, 13.7 to 16.9% from wave I to III. Overall, across the waves, the proportion of EBF up to 6 months among the mothers in the low wealth quintile was 4, 9 and 42%, and 7, 26 and 15% for the ones in the highest wealth quintile. The proportion of EBF up to 6 months has been increasing among HIV positive mothers while fluctuating among their counterparts across the waves.

**Conclusion:**

The proportion of EBF up to 6 months has been increasing in the Moshi municipality but is below the national average. While establishing trends of EBF at the national level is commendable, research to establish trends over smaller geographical areas is needed to provide a true picture that may otherwise be masked.

## Introduction

There is ever growing evidence of the benefits of breastfeeding (BF) to the child and the mother [1]. The short term benefits to the child include the prevention of infections, such as acute respiratory infection and diarrhoea [1], which are among the leading causes of child mortality in low and middle income countries. Breastfeeding also provides long term benefits including the potential to prevent Type II diabetes and obesity [2]. Among all interventions that can reduce under-five mortality from preventable causes, optimal breastfeeding was found to lead, with a potential to prevent up to 13% of this mortality [3]*.* The benefit was found to increase in proportion to the duration of breastfeeding.

The World Health Organization (WHO) recommends all children are exclusively breastfed (EBF). It has been recommended to have a target of 90% of infants being exclusively breastfed for the first six months of life [1, 3]. However, to date, only 43% are exclusively breastfed globally and no country has reached the target proportion of 90%, with Rwanda having the highest proportion (85%) of EBF [1]. It is important to be able to monitor the proportion of EBF and its changes over time towards meeting the set target.

The majority of countries, Tanzania included, have reported trends of EBF at the national level, but not at regional (smaller geographical) levels. However, national estimates may mask the true picture in the regions. With some studies reporting regional variability of EBF [4, 5], the need to monitor the trend of EBF at the regional level cannot be overstated. The proportion of EBF is determined by socio-economic and demographic factors, which may vary considerably from one region to another. This study was carried out to determine the trend of EBF in the Moshi municipality in northern Tanzania from 2002 to 2014. The findings will give a picture of EBF at the regional level and of different sub-groups of mothers in the community. This understanding is crucial to enable health personnel at the national, regional, district and health facility level to develop interventions as per their scope.

## Methods

### Data source

The data for this study came from three open cohorts of women attending the Majengo and Pasua health centres in Moshi Municipality, Kilimanjaro region Tanzania [6–8]. To refer to these three distinct groups of women recruited in the parent study, the term “wave” will be used. Wave I for those who delivered between 2002 and 2004, wave II between 2005 and 2012 and wave III between 2013 and 2014, irrespective of when they were recruited into the cohort. Women were recruited between 2002 and 2004 and again in 2012–2014, primarily to evaluate the prevalence of HIV and sexually transmitted infections (STI) among pregnant women in the third trimester. Between 2005 and 2011, only HIV positive women were recruited. As some HIV negative women recruited up to 2004 may have delivered in 2005, their feeding practices are reported as wave II results. The same data collection and follow-up methods were used across all waves.

The women were followed up until delivery, during puerperium and afterwards, as long as they stayed in the cohort. After being seen at delivery, each mother-infant pair was seen at the centres at 1, 3 and 6 months and continued with visits until the child was 60 months old. Trained nurses carried out face to face interviews using questionnaires with structured and non-structured questions. During the visits, information on infant and young feeding practices, family planning use and growth monitoring data for children was collected. The information on infant and young child feeding that was collected included initiation of breastfeeding, colostrum giving, the practice of EBF, and complementary feeding. This paper analyses the data on EBF practices to give the trends of EBF. The practice of EBF was determined by asking at each visit whether the mother had fed her child anything other than breast milk since birth or the last clinic visit and, what she had introduced and how old the child was at the time. This information was then used to determine the age at which EBF ceased.

### Study area

Data was from cohorts established in the Moshi Municipality in the Kilimanjaro region. In 2015–16, Kilimanjaro had a population of 1,560,354 with 285,263 being women of reproductive age (15–49 years) [9]. A large majority of pregnant women (98.3%) in Kilimanjaro region attend antenatal clinic care (ANC) at least once during their pregnancy and 87.6% have health facility deliveries [9]. This is very similar to the estimated national average for urban residents who attend ANC at least once of 98.4 and 81.6% who have health facility delivery [9].

### Study design and population

Using the extracted data, a longitudinal study was carried out by following-up a mother and her infant from delivery up to when the infant was six months old. Any mother-infant pair with no information on initiation of breastfeeding or when other foods were introduced was excluded from the study. This is because the dependent variable was defined using these two variables.

### Sample size

All eligible mothers in the cohort were included in this proposed study. Data from Demographic and Health Survey (DHS) reports over the study period (2002–2014) have shown an increase of 9% in the proportion of EBF among infants of 0–6 months at the national level from 41% in 2004/5 to 50% in 2014 [9]. Assuming a similar proportion in the studied area, and using a STATA (Stata Corporation, College Station, TX, USA) version 13 command;

*Power two proportions p1 p2, n1() n2 ()* (*p1 = 0.41*, *p2 = 0.5, n1 = 1656, n2 = 403).*

With 1656 women in wave I, and 403 in wave III, the study has 90% power to show a difference of 9% in the proportion of women who exclusively breastfed infants under 6 months between the two groups, as significant at the 5% level.

### Data analysis

Data was analysed using STATA Corporation, College Station, TX, USA version 13. Exclusive breastfeeding was categorized based on the World Health Organization (WHO) definition of EBF “The receipt of only breast milk (either directly from the breast or expressed); only oral rehydration solution, drops, and syrups (vitamins, minerals, or medicines) are permitted for the first 6 months of life” [10]. Infants who were exclusively breastfed up to 30 days or more were classified as exclusively breastfed up to 1 month. Infants who were exclusively breastfed up to 90 days or more, were classified as exclusively breastfed up to 3 months. And those who were exclusively breastfed up to 180 days were classified as exclusively breastfed up to 6 months. Breastfeeding practices were analysed using the Chi-Square test to evaluate if there was a statistical difference in the proportion of those exclusively breastfed in each duration category (1, 3 and 6 months) across the waves. A Chi-square test for a linear trend in changes in exclusive breastfeeding up to 6 months across the three waves was carried out. All *p value* were derived from two-sided tests and a *p value* of less than 0.05 was considered statistically significant. Trends for wave I, wave II and wave III were established and compared. Also, separate proportions were made for HIV positive mothers and those that were negative, and for the ones in the highest and lowest wealth quintiles.

Wealth quintile was established using principle component analysis (PCA) [11]. The variables that were selected for Socioeconomic Status(SES) estimation were; employment status, level of education, travelling habits, income per month, house ownership, type of walls in a household, sanitation facility and source of water supply. The variables that had missing data was replaced by the mean. A total of 19 parameters were used to estimate the wealth quintile of the mothers. Wealth quintile was then categorized into 5 equal categories.

### Ethical considerations

Ethical clearance certificate number 2020 was obtained from the College Research and Ethical Review Committee (CRERC) of Kilimanjaro Christian Medical University College. Permission to use the dataset was provided by the supervisor of the cohort. Mothers provided informed written consent and assented for their children to take part in the cohort. Mothers were informed that their information may be used for research purposes. Confidentiality and anonymity has been maintained whereby this study only accessed and used coded data without individual identifiers.

## Results

### Participants’ flow

**Fig. 1:**
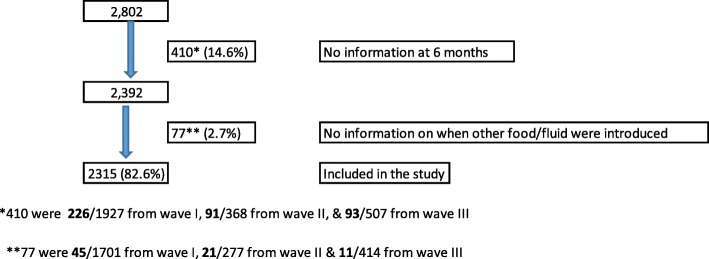
Selection of women included in the study

Information was available for 2802 mother-infant pairs. Out of these, 410 (14.6%) were excluded because the mother-infant pair was not followed up to the age of six months and 77 (2.7%) missed information on when food or fluid were introduced. A total of 2315 (82.6%) mother-infant pairs met the inclusion criteria (Fig. 1).

### A description of baseline characteristics of mothers and infants in the cohort

The median age of the mothers was 25 (Interquartile range (IQR) 21, 29) years. Most of the mothers (73.3%; *n* = 1676) had primary level education and 67.6% (*n* = 1376) had a parity of 2–4 children. The majority of women were HIV-negative *n* = 1971 (87.1%). A large proportion of the mothers *n* = 1656 (71.5%) came from wave I, while *n* = 256 (11.1%) were from wave II and *n* = 403 (17.4%) from wave III. The waves were different in the following characteristics; age of the mother, HIV status, parity and education of the mother (*p value* < 0.05), while similar in marital status and mode of delivery (*p value* > 0.05), (Table 1).

**Table 1:** Baseline characteristics of mothers in the Moshi Municipality Cohort (N = 2315)

Characteristics	Total (*N* = 2315)	2002/4 (*n* = 1656, 71.5%)	2005/12 (*n* = 256, 11.1%)	2013/14 (*n* = 403, 17.4%)	χ2	*P* value
	*n* (%)	*n* (%)	*n* (%)	*n* (%)		
Age of the mother(years) (*n* = 2286)					97.971	< 0.001
≤ 24	1132 (49.5)	905 (55.1)	54 (22.4)	173 (43.0)		
> 24	1154 (50.5)	738 (44.9)	187 (77.6)	229 (57.0)		
HIV status (*n* = 2263)					668.41	< 0.001
Negative	1971 (87.1)	1535 (94.9)	87 (35.5)	349 (87.0)		
Positive	292 (12.9)	82 (5.1)	158 (64.5)	52 (13.0)		
Education of the mother (n = 2286)					1226.27	< 0.001
No formal education	87 (3.8)	82 (5.0)	4 (1.7)	1 (0.2)		
Primary education	1676 (73.3)	1419 (86.4)	215 (89.2)	42 (10.4)		
Secondary and higher	523 (22.9)	142 (8.6)	22 (9.1)	359 (89.3)		
Marital status(*n* = 2292)					4.0477	0.4
Married/cohabiting	2095 (91.4)	1508 (91.5)	228 (93.8)	359 (89.5)		
Single	173 (7.5)	122 (7.4)	13 (5.3)	38 (9.5)		
Once married	24 (1.1)	18 (1.1)	2 (0.8)	4 (1.0)		
Parity (*n* = 2160)					209.582	< 0.001
Multiparous [2–4]	1460 (67.6)	980 (59.7)	218 (89.7)	262 (94.9)		
Nulliparous	654 (30.3)	628 (38.3)	17 (7.0)	9 (3.3)		
Grand multipara (> 4)	46 (2.1)	33 (2)	8 (3.3)	5 (1.8)		
Mode of delivery (*n* = 2265)					2.752	0.252
Vaginal delivery	2126 (93.9)	1522 (94.1)	237 (95.2)	367 (92.2)		
Caesarean section	139 (6.1)	96 (5.9)	12 (4.8)	31 (7.8)		

The majority (91.2%; *n* = 2112) were seen by the recruiter for only one pregnancy. There was almost an equal proportion of male (51.1%) and female (48.9%) infants. There were 26 (1.1%) twins, 32 (1.4%) preterm, and 646 (27.9%) born with a weight less than 2500 g i.e. low birth weight, (not shown).

### Trend of exclusive breastfeeding over years

**Fig. 2:**
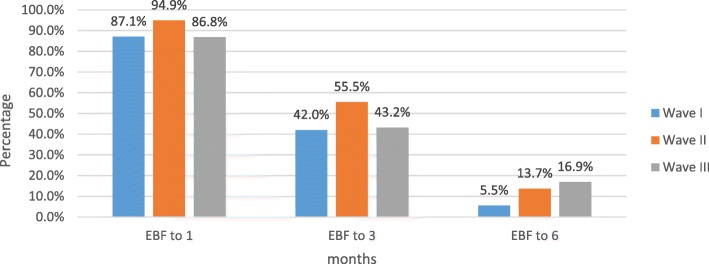
Trend of exclusive breastfeeding over the three waves [*N* = 2315 wave I *n* = 1656, wave II *n* = 256, wave III *n* = 403]

Overall, the proportion of infants who were exclusively breastfed up to 6 months increased from 5.5% in wave I to 13.7% in wave II to 16.9% in wave III. There was a statistically significant linear trend of EBF between the three waves with a χ2 = 63.4 and *p value* < 0.001, (Fig. 2).

A further analysis showed that the proportion of infants who were exclusively breastfed in all waves was higher at 1 month and continued to drop as an infant aged. The proportion of EBF at 1 month was the highest in wave II (94.9%). For EBF up to 3 months, the proportion ranged from 42 to 55.5% being again highest in wave II. The proportion of EBF up to 6 months was below 20% in all waves. There was a statistically significant association between the different waves and the proportion of exclusive breastfeeding at the different time durations (χ^2^ = 13.2 *p value* = 0.001 up to 1 month, χ2 = 16.5, *p value* < 0.001 up to 3 months and χ2 = 65.2 *p value <* 0.001 up to 6 months), (Fig. 2).

### Trend of exclusive breastfeeding by wealth quintile

**Fig. 3:**
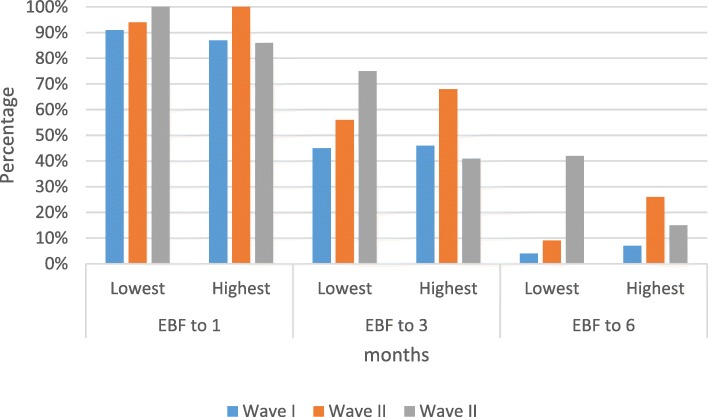
Trend of exclusive breastfeeding practices between those with lowest and highest wealth quintile, 2002–2014. [N = 2315 lowest wealth quintile *n* = 473, highest wealth quintile *n* = 461]

The proportion of EBF among mothers of lowest wealth quintile increased across the waves at each time unit. Looking at the infants who were breastfed up to 6 months, the proportion of EBF among those of lowest wealth quintile increased from 4 to 9% to 42% across the three waves. Except for up to 1 month, there were significant differences in the proportion of EBF across waves among women with the lowest wealth quintile (χ^2^ = 2.28 */p value* = 0.319 up to 1 month, χ^2^ = 7.56 *p value* = 0.023 up to 3 months and χ^2^ = 29.76 *p value <* 0.001 up to 6 months), (Fig. 3).

Among women in the highest wealth quintile, those in wave II had the highest proportion of EBF at any time point. Looking at 6 months, the proportion changed from 7, to 26% to 15% from wave I to wave III. For mothers of the highest wealth quintile, except for the up to 1 month observation, there were significant differences in the proportion of EBF across waves (χ^2^ = 3.07 *p value* = 0.216 up to 1 month, χ^2^ = 6.13 *p value* = 0.047 up to 3 months and χ^2^ = 5.98 *p value <* 0.05 up to 6 months), (Fig. 3).

### Trend of exclusive breastfeeding by HIV status

**Fig. 4:**
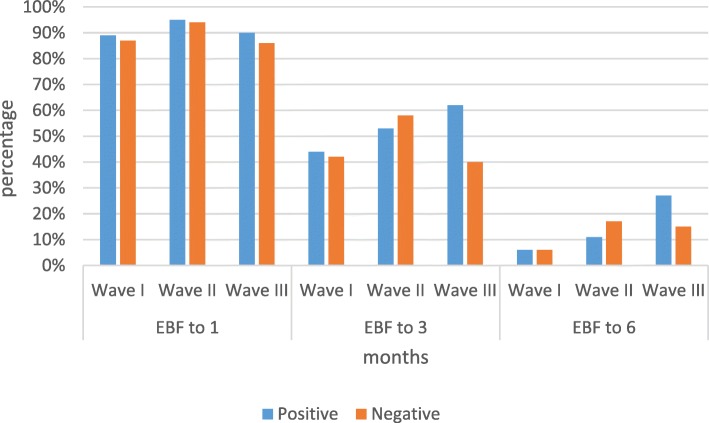
Trend of exclusive breastfeeding practices based on HIV status of the mothers, 2002–2014. [N = 2315 HIV + ve *n* = 292, HIV –ve *n* = 1971]

At 6 months, the proportion of EBF among HIV positive mothers increased from 6 to 11% to 27% across the waves. The significant difference in the proportion of EBF across the waves was only at 6 months (χ^2^ = 3.10 *p value* = 0.212 up to 1 month, χ^2^ = 4.14 *p value* = 0.126 up to-3 and χ^2^ = 13.56 *p value <* 0.001 up to 6). On the other hand, among HIV negative mothers, the proportion of EBF up to 6 months increased from 6% in wave I to 17% in wave II, then dropped to 15% in wave III. Among HIV negative mothers, the great majority, these changes in the proportion were statistically different at 3 and 6 months (χ^2^ = 4.19 *p value* = 0.123 up to1 month, χ^2^ = 8.94 *p value* = 0.011 up to3 months and χ^2^ = 47.49 *p value <* 0.001 up to 6 months). Except for wave II, at whatever time unit, the proportion of EBF among HIV positive mothers was higher. During wave I, the proportion of EBF among HIV positive and negative mothers was the same at 6 months, (Fig. 4).

## Discussion

This is the first study in Tanzania to establish the trend of EBF at a regional level over a period of 12 years. Although the proportion of EBF up to 6 months has been increasing overall across the years, it was not consistent across all the sub-groups of women. The proportion of EBF to 6 months showed the greatest increase among women of the lowest wealth quintile and those that are HIV positive, while there has been no significant increase among their counterparts. However, the general trend and that observed in all sub-groups of mothers is the decrease in EBF with an increase in infant age.

The overall trend of EBF up to 6 months in Moshi municipality has been observed to increase across the years. There has been a reported increase in the national average proportion of EBF in Tanzania [9] and Kenya [12], while in the neighboring countries (e.g. Uganda) the proportion of EBF has been fluctuating [13–15]. Although it is encouraging to observe that the trend in Moshi municipality has been increasing across the years, the proportion of infants EBF up to 6 months is well below the recommended target of 90% [1]. Efforts to improve the proportion of EBF of infants under 6 months need to be emphasized in Tanzania and perhaps regional estimates taken as the guide to set targets.

The differences in proportions from the national average may be due to intrinsic differences between regions in a vast country like Tanzania. Other studies such as those conducted in Brazil [16], India [17], Nepal [5] and Nigeria [4] have reported regional variability. Kilimanjaro is one of the few regions in the country where the socio-demographic characteristics and the availability of well-being services are almost the same in rural and urban areas [9]. These characteristics are comparable to the national average for urban settings [9]. Other cross-sectional studies in other regions have also shown the proportion of EBF of infants 0–6 months to be different from the national average [18, 19]. While providing national estimates of the proportion of EBF of infants 0–6 months is crucial [1], efforts to provide regional estimates may help to better understand the situation on the ground which may be different from the national status.

Another reason for the difference observed in the proportion of EBF of infants under 6 months may be due to differences in the study design [20] whereby this study used a cohort while estimates provided by DHS use a cross-sectional study design. Khanal et al. (2016) observed that cohort studies would provide lower proportions of EBF compared to cross-sectional studies although probably more ‘realistic’ proportions of EBF [20]. The proportions in this study are comparable to estimates in another cohort study in Nepal (8.9% EBF among infants up to 6 months, 2014) [20] and from the latest estimates made by DHS in the region (14.6% EBF among children 0–6 months 2015–16) [9].

A drop in EBF as infants age has been reported in global estimates [21], and in Brazil [16], India [17], Nepal [5], Nigeria [4] and Tanzania [9]. In this study, the proportion of EBF dropped by almost half from 1 month to 3 months. Understanding the point where there is a significant drop in EBF may guide the focus of interventions. For example, in Nepal the proportion of EBF among infants aged 0 up to 6 months increased mainly by increasing the proportion of those who were exclusively breastfed at 4 months [5]. The current vaccination schedule in Tanzania includes vaccines offered at six weeks after delivery. With an overall vaccination coverage of 75% in Tanzania and 93% in Kilimanjaro region [9], this contact with the health facility may be used as an opportunity to strengthen interventions. The counselling that mothers receive during health facility visits has shown to improve EBF practices [4, 9, 22, 23] and particularly when provided after delivery [18].

We argue that Tanzania as a country has enough potential to do both make studies for trend of EBF at smaller geographical areas and establish satellite cohorts for EBF estimates. The latest DHS for the first time presented regional estimates of EBF [9], and showed regional variabilities. There are three schools of public health located in the northern, eastern and north-west areas of the country. Students at these institutions are required to conduct studies to graduate, and they have been doing this. This shows that there are enough resources (human, financial and time) and potential to conduct studies. A consideration may be made to make EBF related research a priority. Also, there are implementation partners (non-governmental organizations) all over the country. Taking the magnitude of the importance of EBF on the general well-being of the population, it may be important to give EBF a top priority for their interventions and explorations.

Making estimations at a global level, Victoria et al. (2016) found that the trend for EBF was increasing over years, the rate was higher among those with the highest wealth quintiles compared to those with the lowest [1]. With Sustainable Development Goal targeting equity [24], it is heartening to observe that, in the studied population, those of the lowest wealth quintile are doing well in this highly beneficial practice. However, caution has to be taken while looking at this. Studies have shown that the poor may tend to breastfeed for longer as they have nothing else to feed their infants, coined ‘reverse causality’ [25]. Ultimately, the infants do not benefit from the practice if they are EBF beyond 6 months. Other than that, it has been observed that ultimately the ones with the highest wealth quintile would adopt the recommended health beneficial practices. Efforts should be made to ensure that in Tanzania all women, despite their wealth quintiles, adopt and practice EBF.

Recommendations on EBF among HIV positive mothers have varied over the years [26] guided by research results, basically because of the fear of transmitting the infection through breast milk. In 2002–2004, the recommendation started with EBF for 3 months followed by abrupt cessation, but the current recommendation is using anti-retroviral treatment and breastfeeding the infants exclusively for the first six months of life followed by continued breastfeeding up to twelve months [27]. In this context, it is encouraging that the proportion of EBF among HIV positive mothers is higher than their negative counterparts. However, this is not surprising given that in the current PMTCT program, HIV-positive pregnancy women and postpartum women are seen monthly for counselling and care, including counselling on breastfeeding and infant feeding practices. Nevertheless, the proportion of HIV-positive mothers EBF in wave III were still lower than the recommended proportions. The proportion of EBF reported in this study among HIV positive mothers are similar to the proportion of a study carried out in the eastern part of the country (proportion of EBF up to 6 months 13.3%) in 2010 [28], but lower than that reported in a recent study carried out also in the same area (proportion of EBF among children 0–6 months 77%) in 2016 [22]. The differences in the proportion of EBF with those observed in the recent study may have resulted from using different study designs. The differences may also have been because of different settings. With the change in EBF policy among HIV positive mothers, the adoption may be faster in the sites used in the recent study. Authors in the article observed that providers in one of the facility were probably exposed to more trainings [22] which might have made providers more aware of recommendations. However, this referred study [22] shows that it is possible to increase the proportion of EBF among HIV positive mothers and strategies to meet this are to be strengthened.

The limitations for this study include being a hospital based study of a cohort with a loss to follow-up of about 20%. This may introduce selection bias, whereby those who stayed with the cohort for at least 6 months after delivery may be different from those that were lost. Information bias was limited by using a standardized questionnaire and training the research assistants. Establishing EBF by making a follow-up of feeding practices from delivery until the child was six months of age, rather than a 24-h recall, is among the strength of this study [20].The findings are to be interpreted with caution; wave II contains more HIV positive mothers as the cohort recruitment focused on them and hence over representation of HIV positives in the whole cohort.

## Conclusion

While establishing trends of EBF at a national level is commendable, efforts to establish the trend in the smaller geographical areas are needed to provide a true picture that may otherwise be masked and which is necessary to guide localized interventions. The trend may also need to be explored for women of different socio-economic and health status. Interventions such as counselling should focus on mothers with infants of one month of age.

## Abbreviations

ANC: Antenatal clinic care
CRERC: College Research and Ethical Review Committee
DHS: Demographic Health Survey
EBF: Exclusive breastfeeding
IQR: Interquartile range
PCA: Principle Component Analysis
STI: Sexually Transmitted Infections
